# Index to Original Communications in the Medical Journals of the United States and Canada for 1877

**Published:** 1879-03

**Authors:** 


### BIBLIOGRAPHICAL.

Index to Original Communications in the Medical Journals of the United States and Canada for 1877. Classified by subjects and authors. Compiled by Wm. D. Chapin, N. Y. Price $1.

This little volume of forty-seven closely-printed pages will be found a convenient reference book to those who may desire to refer to some particular article. In addition to the United States and Canada journalistic literature, the "Index" of 1878, now in active preparation, will embrace that of Great Britain and Ireland. This addition will not increase the price, which will remain one dollar.

